# X-ray nanodiffraction analysis of stress oscillations in a W thin film on through-silicon via

**DOI:** 10.1107/S1600576715023419

**Published:** 2016-02-01

**Authors:** J. Todt, H. Hammer, B. Sartory, M. Burghammer, J. Kraft, R. Daniel, J. Keckes, S. Defregger

**Affiliations:** a Department of Materials Physics, Montanuniversität Leoben, Leoben, Austria; b Materials Center Leoben Forschung GmbH, Roseggerstrasse 12, Leoben, Austria; c European Synchrotron Radiation Facility, 6 Rue Jules Horowitz, Grenoble, France; d ams AG, Unterpremstätten, Austria; e Department of Physical Metallurgy and Materials Testing, Montanuniversität Leoben, Austria

**Keywords:** X-ray nanodiffraction, residual stress, through-silicon via

## Abstract

Synchrotron X-ray nanodiffraction is used to analyse residual stress distributions in a 200 nm-thick W film deposited on the scalloped inner wall of a through-silicon via. The diffraction data are evaluated using a novel dedicated methodology which allows the quantification of axial and tangential stress components under the condition that radial stresses are negligible. The results reveal oscillatory axial stresses in the range of ∼445–885 MPa, with a distribution that correlates well with the scallop wavelength and morphology, as well as nearly constant tangential stresses of ∼800 MPa. The discrepancy with larger stress values obtained from a finite-element model, as well as from a blanket W film, is attributed to the morphology and microstructural nature of the W film in the via.

## Abstract 2

X-ray nanodiffraction is used to evaluate axial and tangential residual stress distributions in a W thin film deposited on the scalloped inner wall of a through-silicon via. The results reveal oscillatory stress distributions which correlate well with the scallop wavelength and morphology.

## 1. Introduction   

X-ray diffraction is routinely used to evaluate X-ray elastic strains in engineering polycrystalline materials and, by applying appropriate X-ray elastic constants (XECs), to quantify residual stresses (Noyan *et al.*, 1995 ▸). In the past few years, there has been enormous progress in the characterization of strongly inhomogeneous residual stress distributions in thin films and near-surface regions using grazing-incidence techniques (Genzel *et al.*, 2011 ▸; Angerer & Strobl, 2014 ▸) and scanning transmission techniques (Keckes *et al.*, 2012 ▸; Vaxelaire *et al.*, 2014 ▸). Cross-sectional X-ray nanodiffraction operating with beams down to 50 nm in diameter has been extensively used to analyse the correlation between microstructure and stress in nanocrystalline thin films using a common wide-angle X-ray diffraction geometry (Bartosik *et al.*, 2013 ▸; Stefenelli *et al.*, 2013 ▸). Currently, it is still challenging to analyse stress distributions in small technological components with complex geometry used, for example, in microelectronics (Noyan *et al.*, 2004 ▸). The main challenges here are (i) the need for a position-resolved characterization and (ii) usually complex stress fields whose quantification from the measured data is not trivial.

Three-dimensional integration of microelectronic chips is an emerging technology which is based on multi-level integration of functional components by vertical stacking and connecting of die structures (Knickerbocker *et al.*, 2008 ▸). A conductive connection between the circuits on each side of a wafer is achieved by through-silicon vias (TSVs) (Sakuma *et al.*, 2008 ▸), which are etched into the dies in the form of microscopic channels and then filled with a TSV metallization stack including copper or tungsten. In the case of TSVs with large diameter, a deposition of a thin metallization layer on the TSV sidewall is sufficient.

During metal deposition, intrinsic stresses may form in the metal (Daniel *et al.*, 2010 ▸). Their magnitude and sign depend decisively on the process conditions. Because of the mismatch of the coefficients of thermal expansion (CTE) between metal and silicon, relatively high tensile thermal stresses form in the metal after cooling from the deposition temperature to the operating temperature (Eiper *et al.*, 2007 ▸). This may result in the initiation of micro-cracks (Liu *et al.*, 2013 ▸), which can modify the device characteristics and even result in structural damage of the three-dimensional structures (Ranganathan *et al.*, 2008 ▸). It is therefore important to develop a methodology to quantify the magnitude of residual stresses in TSVs in order to (i) correctly interpret the reliability issues and (ii) subsequently optimize the production route.

Recently, several techniques have been used to determine multiaxial strain–stress fields in TSV structures. These include especially micro-Raman spectroscopy (Liu *et al.*, 2009 ▸) and synchrotron white-beam micro-Laue X-ray diffraction (XRD) (Liu *et al.*, 2014 ▸; Budiman *et al.*, 2012 ▸; Nakatsuka *et al.*, 2011 ▸; Sanchez *et al.*, 2014 ▸), which were used predominantly to quantify strain–stress fields in silicon. It has been observed that the highest stress concentrations in silicon wafers are at the metal/wafer interfaces and that the stress magnitude decreases on a length scale of a few micrometres from the TSV wall. Complementary to this, residual stresses in Cu and W films were analysed using laboratory (Krauss *et al.*, 2013 ▸) and synchrotron XRD (Budiman *et al.*, 2012 ▸), whereby stress magnitudes up to a few hundred MPa were reported.

The ongoing effort for the miniaturization of microelectronic devices requires the application of cutting-edge analytical techniques which allow for very local characterization of residual stress fields in the interconnect structures. The aim of this work is (i) to derive a methodology for the evaluation of axial and tangential stress fields in TSV metallization from two-dimensional X-ray nanodiffraction data, (ii) to quantify the stresses in a scalloped 200 nm-thick W film deposited on the inner wall of a TSV, and (iii) to correlate the stress variation with numerical simulations based on a finite-element (FE) model.

## 2. Experiment   

**Figure 1:**
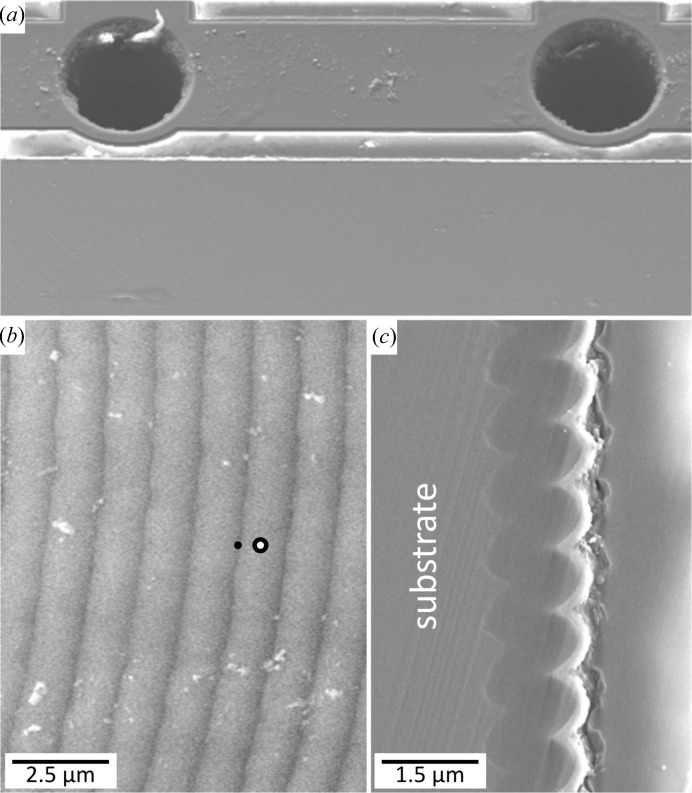
Scanning electron micrographs of the TSV. The circular via had a diameter of 100 µm (*a*). The details of the inner wall surface (*b*) and cross section (*c*) document the scalloped morphology of the silicon substrate and the stack of SiO_2_, W and Si_3_N_4_ sublayers. The filled and open circles in (*b*) schematically indicate the positions of the W film scallop hill and valley at which X-ray nanodiffraction data from Fig. 3 ▸ were collected.

The TSV sample in this study was a blind via (Kraft *et al.*, 2011 ▸) with a diameter of 100 µm and an axial length of 250 µm. The via was etched using a deep reactive ion-etching process [into a 250 µm Si(100) wafer, which was bonded to a 725 µm-thick Si handling wafer] resulting in the formation of a scalloped wall (Krauss *et al.*, 2013 ▸) which was afterwards coated with a dedicated stack of SiO_2_/W/SiO_2_/Si_3_N_4_ (Fig. 1 ▸). The 200 nm-thick W sublayer, the only crystalline feature of the stack, was deposited using chemical vapour deposition at 673 K.

**Figure 2:**
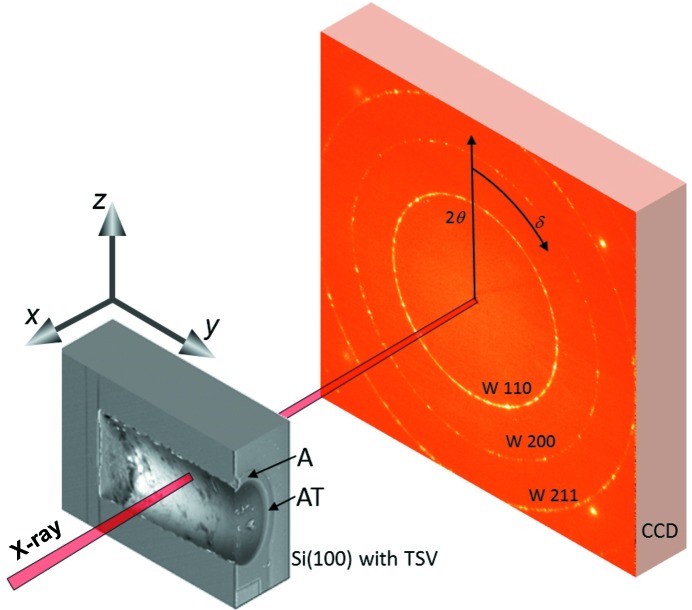
A schematic description of the X-ray nanodiffraction experiment performed using a beam of 100 nm in diameter on an isolated TSV in transmission geometry. Two scans along the *y* axis were performed at the *z* positions denoted as A and AT, with a step of 100 nm, and the diffraction signal was collected using a two-dimensional detector. θ and δ denote radial and azimuthal positions of the W reflections, respectively.

The transmission X-ray nanodiffraction experiment was performed at the nano-focus extension of beamline ID13 (Keckes *et al.*, 2012 ▸) of the European Synchrotron Radiation Facility (ESRF) in Grenoble, France. The high-brilliance X-ray source and dedicated X-ray focusing optics combined with a nano-positioning sample stage allowed for a spatially resolved residual stress characterization using a beam of 100 nm in diameter and a photon energy of 14.7 keV (Fig. 2 ▸). A FReLoN4M two-dimensional detector was placed 69.8 mm downstream of the sample in order to collect the transmission diffraction signal. For the experiment a dedicated sample with dimensions of ∼140 × 400 × 200 µm in the **x**, **y** and **z** directions (Fig. 2 ▸), respectively, was machined using the focused ion beam (FIB) milling technique. The sample comprised a single TSV where 1/3 of the TSV cylinder was removed by a FIB segmental cut parallel to the cylinder axis in order to prepare a free TSV inner-wall surface for the transmission XRD geometry (*cf.* Fig. 2 ▸). The morphology of the TSV feature was analysed using a dual-beam FIB workstation (AURIGA-CrossBeam from Zeiss). Complementary to the synchrotron nanodiffraction characterization, 200 nm-thick blanket W films deposited on an Si(100) wafer were characterized using a Rigaku SmartLab five-axis diffractometer equipped with Cu *K*α radiation, a parabolic multilayer mirror in the primary beam and a secondary graphite monochromator. In order to interpret the stress behaviour in the TSV structures, an FE simulation was performed using the commercial FE package *ANSYS* (version 14.5). Using the FE model, it was also proved that the cut through the TSV according to Fig. 2 ▸ did not induce any significant stress relaxation in the thin W film.

## 3. Methodology   

Two-dimensional diffraction data collected during the X-ray nanodiffraction experiment from various TSV positions were used to determine the lattice spacing 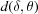 of the W(200) crystallographic planes using Bragg’s law by analysing the Bragg angles θ of W 200 Debye–Scherrer (DS) rings at different azimuthal positions δ (Fig. 2 ▸). For this reason, the collected two-dimensional patterns were treated using the software *Fit2D* (Hammersley *et al.*, 1996 ▸) and 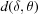 values, which represent film properties averaged along the beam direction in the irradiated volume, were determined for 36 azimuthal δ sections.

Measured X-ray elastic strains in W films can be expressed generally as 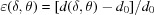, where 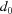 is the W unstressed lattice parameter. For the evaluation, it was supposed that the strain state in W films is triaxial with 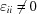, and shear strain components were neglected for simplicity with 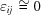. The reasonability of this assumption was verified by the FE model, which also indicated negligible shear strains. The measured strain can then be expressed generally as follows (Stefenelli *et al.*, 2013 ▸):
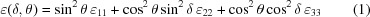
where 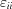 represent the unknown elastic strain components and the indices 1, 2 and 3 correspond to the axes *x*, *y* and *z* in Fig. 2 ▸, respectively. Two axial scans were carried out (by moving the sample along the *y* axis with a step of 100 nm) at two sample *z* positions denoted as A and AT, corresponding to the TSV upper border and the TSV centre, respectively (*cf.* Fig. 2 ▸).

During the data treatment, it was supposed that only axial 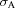 and tangential 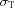 stress components are significant in the W thin film and that the radial stress component can be neglected (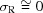) owing to the nearly free TSV inner surface. This crucial assumption was verified by the FE model and the radial stresses were found to be smaller than 50 MPa.

At the measurement position A, the tangential, axial and radial stress–strain directions coincide with the **x**, **y** and **z** vectors (Fig. 2 ▸) and therefore relationships can be expressed as
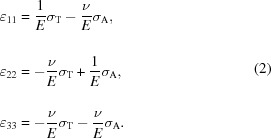
At the measurement position AT, the strains can be expressed as
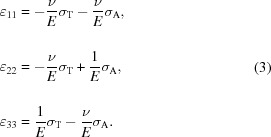

*E* and ν represent Young’s modulus and Poisson’s ratio of tungsten. Inserting equations (2) and (3) into equation (1), the measured strain at the positions A and AT can be expressed as
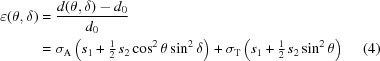
and
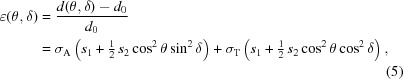
respectively, where 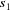 = −7.27 × 10^−9^ Pa^−1^ and 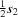 = 33.01 × 10^−9^ Pa^−1^ are the XECs of tungsten (Featherston & Neighbours, 1963 ▸). Tungsten is an elastically isotropic material and therefore crystallographic texture was neglected when evaluating residual stresses from the measured X-ray elastic strains.

Equations (4) and (5) indicate that the distortions of the DS rings 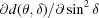 are equal to
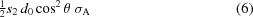
and
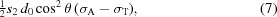
for the A and AT measurement positions, respectively.

## 4. Results   

**Figure 3:**
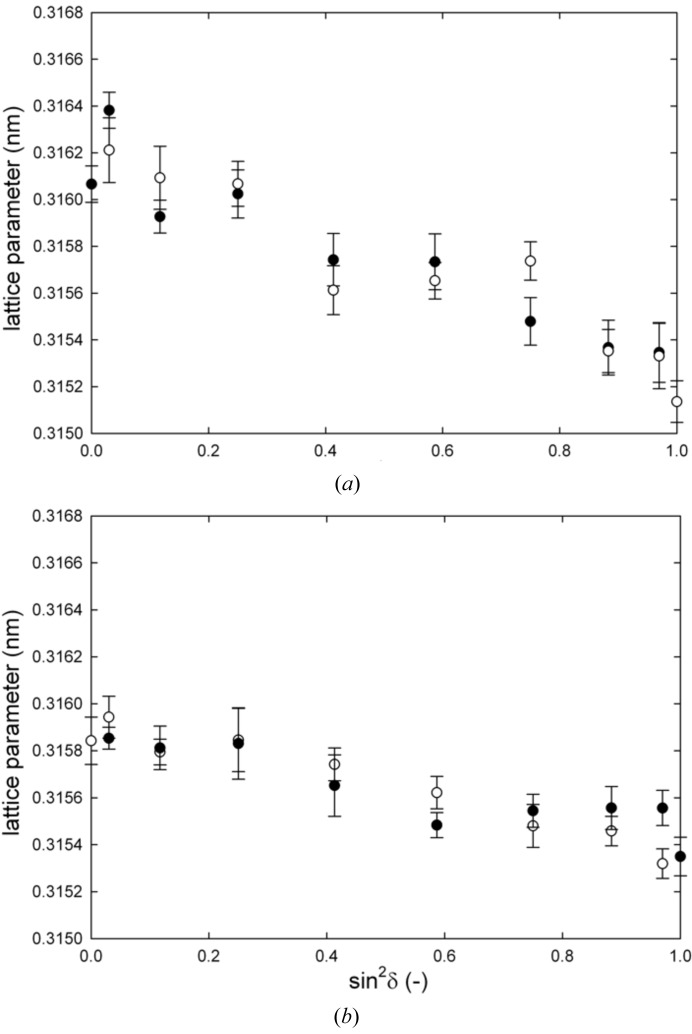
Lattice spacing *d* dependencies on 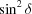 evaluated from two-dimensional data collected during the scanning experiment at the TSV edge, measurement position A, when the X-ray beam hit the via at the W film scallop hill (*a*) and valley (*b*) (*cf*. Fig. 1 ▸ *b*). The different slopes of the dependencies indicate varying magnitudes of residual stresses along the **y** direction. Filled and empty points represent lattice spacing data evaluated for δ azimuthal position intervals of 0–90 and 90–180°, respectively.

Two-dimensional diffraction data collected at various via positions were used to evaluate the lattice spacing 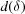. In Fig. 3 ▸, two examples of the lattice spacing dependence 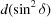 are presented. The data were collected during the scanning experiment at the TSV edge, measurement position A (Fig. 2 ▸), where the X-ray beam hit the via at the W film scallop hill (*cf.* Figs. 1 ▸ *b* and 3 ▸ *a*) and valley (*cf.* Figs. 1 ▸ *b* and 3 ▸ *b*). The different slopes of 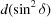 dependencies in Fig. 3 ▸ indicate varying magnitudes of residual stresses along the **y** direction. Similar 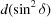 dependencies were constructed from all measured two-dimensional patterns.

**Figure 4:**
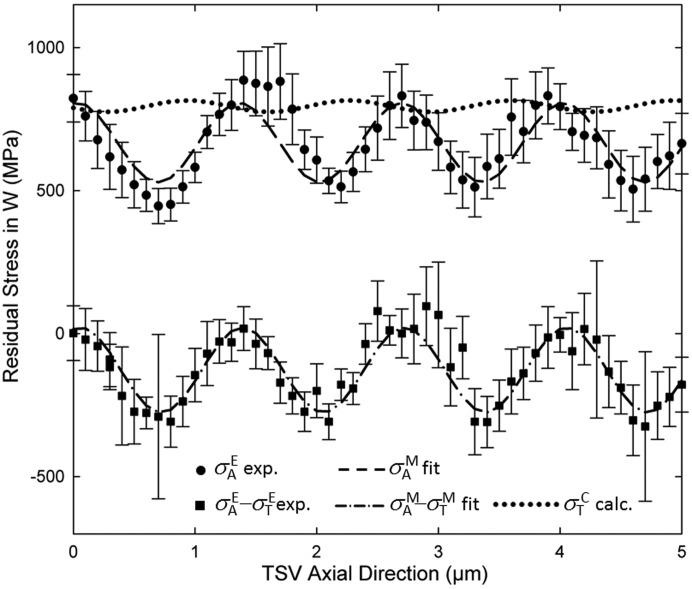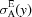 and 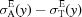 represent experimentally determined dependencies of axial stresses and the difference between axial and tangential stresses, respectively. The data were fitted using sinusoidal functions 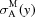 and 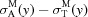 [*cf.* equations (8) and (9)] and the axial dependence of the tangential stresses 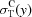 was determined analytically by subtracting the fitted functions.

As a next step experimental (E) dependencies 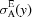 and 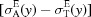 were evaluated from the 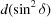 data using equations (6) and (7). These dependencies are presented in Fig. 4 ▸. As the W unstressed lattice parameter, 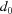 = 0.3158 nm was used, considering the stress-free sample direction as shown in our previous reports (Bartosik *et al.*, 2013 ▸; Stefenelli *et al.*, 2013 ▸), since the TSV was biaxially stressed.

The data in Fig. 4 ▸ document that 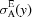 and 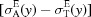 alternate along the *y* axis, the TSV axial direction. For simplicity, the experimental dependencies of the stresses (in MPa) obtained from the two *y*-axis scans at the A and AT positions, 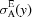 and 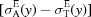, were approximated using the functions
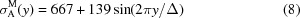
and
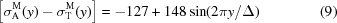
where 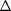 = 1.322 ± 0.12 µm represents the fitted W scallop wavelength which correlates well with the TSV morphology from Figs. 1 ▸(*b*) and 1 ▸(*c*). In order to quantify the tangential stresses 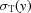, the two fitted functional dependencies 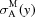 and 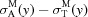 were analytically subtracted and 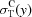 was calculated.

Experimentally determined 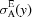, as well as calculated 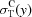 tensile stress dependencies are presented in Fig. 4 ▸. The axial stresses 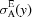 alternate sinusoidally in the range of ∼445–885 MPa, whereas tangential stresses 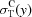 remain relatively constant in the range of ∼770–820 MPa. The sinusoidal dependence of 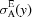 can clearly be attributed to the specific scalloped W film morphology, which induces tensile stress enhancement in the upper thin-film region (closer to the TSV axis) whereas tensile stress minima are located in the curved bottom film regions (farther from the axis). Since in the TSV tangential direction no significant variation of the film morphology takes place, the tangential stresses 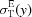 remain approximately constant and tensile at a level of about 800 MPa on average. However, these stress values obtained from the TSV structure are significantly smaller than the equi-biaxial stress values of 1.6 ± 0.2 GPa obtained from the complementary laboratory X-ray diffraction characterization of a blanket W film, in agreement with the data of Krauss *et al.* (2013 ▸). In order to elucidate this discrepancy a comparison with the results from FE simulations was done.

## 5. Discussion   

**Figure 5:**
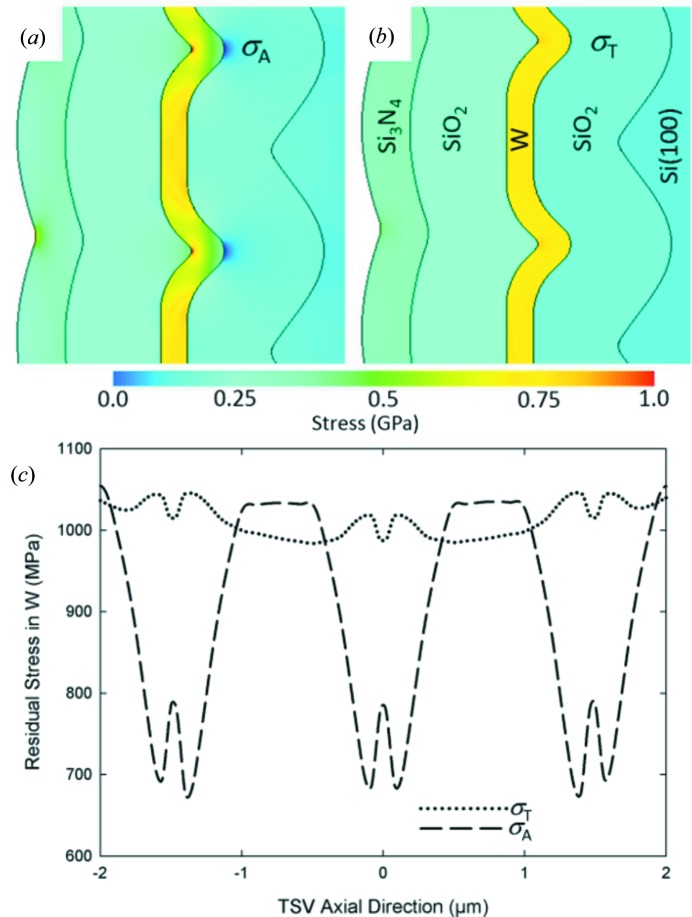
Numerically determined distributions of axial (*a*) and tangential (*b*) stress dependencies at the cross section of a modelled TSV stack document the presence of tensile stresses in the W sublayer. In (*c*) thickness-averaged stress distributions are presented.

The main aim of the FE model was to analyse the distribution of residual stresses in amorphous and crystalline components of the TSV stack structure (Fig. 5 ▸) and to reveal especially the role of the scalloped morphology, deposition temperatures and intrinsic sublayer stresses on the stress distributions. In the model, all materials except tungsten exhibited linear elastic behaviour. For tungsten, a multi-linear stress–strain curve which ceases to be perfectly elastic at the proportionality limit of 1.25 GPa was used. The limit value can be roughly equated with the yield stress.

The parameters for the model were obtained mainly from high-temperature residual stress characterizations of blanket SiO_2_, W and Si_3_N_4_ thin films on silicon using wafer curvature and XRD methods (not presented here). The simulation started with the deposition of the first SiO_2_ sublayer of the stack, which was placed on the scalloped silicon wall of the TSV at a process temperature of 623 K. In an analogous way, all subsequent sublayers of the stack were generated, and the W sublayer was added to the model at 673 K. In the last simulation step, the entire stack on Si was cooled to room temperature. The spatial distributions of axial and tangential stresses are presented in Figs. 5 ▸(*a*) and 5 ▸(*b*). Axial and tangential stress distributions in the modelled W sublayer averaged over the contributions from inner and outer surfaces are presented in Fig. 5 ▸(*c*). The tangential stresses in W remain relatively constant, in agreement with the XRD data. The axial stresses show oscillatory behaviour and a sharp tensile stress increase of about 100 MPa at the bottom of the W sublayer, which can be attributed to the large curvature of the inner surface of this layer.

Similar to the measured stress state in the blanket W film, FE data for a blanket W film model also showed higher tensile stresses, in agreement with the data obtained by Krauss *et al.* (2013 ▸). The smaller stresses from Fig. 4 ▸ could be attributed (i) to the particular rippled inner wall morphology, (ii) to the interaction with other stack layers, as well as to the process sequence, and/or (iii) to significantly different intrinsic stresses formed in blanket and scalloped W films. Deposition kinetics on the scalloped surface of the vertical sidewalls result in the formation of a different film microstructure with relatively small W grains (Krauss *et al.*, 2013 ▸), which in turn decisively influence the intrinsic stress state at the deposition temperature. Consequently, it can be expected that, by optimizing the deposition parameters, higher compressive intrinsic stresses could be generated in W and thus the room-temperature tensile stress could be decreased. Another reason for the smaller stresses could be found in the mechanical conditions in the TSV stack itself (Budiman *et al.*, 2015 ▸). At the stack interfaces, a local delamination might occur, which can lead to a relaxation of thermal stresses caused by the CTE mismatch. The FE data (Fig. 5 ▸) indicate that there may exist stress concentrations, as a consequence of scallop regions with a very high curvature. This might represent a serious reliability issue. Therefore the optimization of the scallop geometry may also serve as an important tool for stress engineering and failure prevention in TSVs.

Methodologically, the results in Fig. 4 ▸ document that even a relatively simple experimental geometry can be used to determine a complex multiaxial stress distribution in tiny microelectronic features. In order to perform this type of analysis, a careful sample preparation, experiment planning and adaptation of the evaluation procedure to the used setup were necessary. One important experimental prerequisite for the use of X-ray nanodiffraction was the fact that the W film was of nanocrystalline nature and, for this reason, the diffraction data collected using a two-dimensional detector (Fig. 2 ▸) exhibited sufficient diffraction statistics.

## 6. Conclusions   

In summary, a new evaluation methodology was used to determine axial and tangential stresses in the W sublayer of a scalloped TSV wall stack, by analysing X-ray nanodiffraction data. The study documents that a relatively simple experimental geometry can be used to evaluate complex submicrometre variations of multiaxial residual stresses in tiny microelectronic features.

The results reveal that the scallops’ morphology decisively influences the local stress state and leads to an axial stress oscillation which, in agreement with FE model results, induces very local tensile stress concentrations. Stress engineering in the TSV metallization can be performed (i) by the optimization of the intrinsic stresses by way of tuning the deposition process, and (ii) by adapting the TSV architecture and morphology.
